# Does gastrostomy make a difference in children with congenital Zika syndrome growth?

**DOI:** 10.1590/1806-9282.20241670

**Published:** 2025-07-07

**Authors:** Hannah Cavalcante Guedes Pinheiro, Mariana Balbino da Silva, Girlene Souza de Azevedo, Thayla Amorim Santino, Maria Carolina Sarmento Campelo, Jousilene de Sales Tavares, Gabriela Lopes Gama, Adriana Melo

**Affiliations:** 1 Professor Joaquim Amorim Neto Research Institute – Campina Grande (PB), Brazil.; 2 Universidade Estadual da Paraíba – Campina Grande (PB), Brazil.; 3 Centro Universitário UNIFACISA – Campina Grande (PB), Brazil.; 4 Universidade Federal de Juiz de Fora – Governador Valadares (MG), Brazil.

**Keywords:** Feeding methods, Microcephaly, Zika virus infection

## Abstract

**INTRODUCTION:**

Children diagnosed with congenital Zika syndrome often experience stomatognathic disorders that may compromise caloric intake via oral feeding

**OBJECTIVE:**

The aim of this study was to assess the effects of gastrostomy on the growth and nutritional parameters of children with congenital Zika syndrome.

**METHODS:**

This prospective cohort study included children with congenital Zika syndrome who underwent gastrostomy and were followed up by the Center of Support for Children with Microcephaly in the state of Paraiba, in northeast Brazil. The study was conducted between April 2019 and May 2023. Children were assessed for growth and nutritional parameters using anthropometric measurements (weight, length, and body mass index) and body composition (body fat) around 1 year before gastrostomy (T0), up to 3 months before gastrostomy (T1), and around 1 year after gastrostomy (T2).

**RESULTS:**

A total of 18 children with a mean age of 47.2±18.7 months at the time of gastrostomy were assessed. Significant improvements were observed in weight-for-age (T1=-3.0±1.3 and T2=-2±1.9; p<0.05) after gastrostomy. However, 11.1 and 38.9% of the children remained below or very below the expected weight-for-age, respectively.

**CONCLUSION:**

Although gastrostomy may increase the anthropometric measures of children with congenital Zika syndrome, this increase may not be enough to achieve adequate weight.

## INTRODUCTION

The first cases of active Zika virus (ZIKV) infections were confirmed in Brazil in 2015, and their association with brain malformations in fetuses was later confirmed by ZIKV detection in the amniotic fluid of two women with a history of rash during pregnancy. These malformations were later described as characteristic of congenital Zika syndrome (CZS), which presents multisystemic clinical signs, such as delayed neurodevelopment and growth and impaired stomatognathic and urinary functions and muscle tone^1,2^.

Stomatognathic disorders may be associated with the inability to obtain the minimum energy requirements via oral feeding. For example, dysphagia and oral motor, sensory, and behavioral disorders are deficits often observed in children with neurological impairments^3,4^, which may result in malnutrition, growth disorders, and micronutrient deficiencies^4,5^. In this sense, alternative feeding methods (e.g., nasogastric tubes and gastrostomy) may reduce the impairments from oral feeding^6,7^. Studies have suggested that gastric feeding may increase weight, height, and skinfold thickness, reduce the time needed for diet administration, and improve the quality of life of children with neurodevelopmental disorders and their caregivers, being an important alternative to prevent malnutrition^5,8,9^.

Although the multisystemic impairments experienced by children with CZS have been previously discussed, gaps in the natural history of this condition and the required nutritional support still need to be clarified. Considering this need and the growth and developmental impairments in children with CZS^10^, the present study aimed to assess the effects of gastrostomy on growth and nutritional parameters in this population. Also, this study aimed to clarify whether children with CZS benefit from gastrostomy as children with other neurological impairments and to support the clinical decision-making regarding the use of this feeding method.

## METHODS

This prospective cohort study was conducted between April 2019 and May 2023 at the Center of Support for Children with Microcephaly linked to the Professor Joaquim Amorim Neto Research Institute (IPESQ) in Campina Grande (Paraiba), Brazil.

The study was approved by the Research Ethics Committee of Alcides Carneiro University Hospital (no. 2.839.838 and CAAE: 91054418.1.0000.5182), followed the Declaration of Helsinki, and was reported following the Strengthening the Reporting of Observational Studies in Epidemiology (STROBE) guidelines.

### Sample

The sample consisted of children diagnosed with CZS who underwent gastrostomy and were followed up by the IPESQ for at least 1 month before and 1 year after the procedure. Children whose data were not recorded before or after gastrostomy or who died within the first year after the procedure were excluded.

The CZS diagnosis was based on the results of reverse transcription-polymerase chain reaction (RT-PCR) tests, obstetric ultrasound, transfontanellar ultrasound, computed tomography (CT), or magnetic resonance imaging (MRI) performed during the first months of life. Neuroimaging abnormalities considered indicative of CZS included calcifications at the gray–white matter junction and any degree of delayed cortical development, ranging from mildly simplified gyral pattern to more severe abnormalities such as lissencephaly, pachygyria, or cortical development malformations^11–13^.

### Assessment and data collection

Caregivers were interviewed, and clinical records from the IPESQ were analyzed for data collection, including caregiver education and socioeconomic status, pregnancy (gestational age and ZIKV infection symptoms), birth (type of birth, presence of arthrogryposis, weight, length, and head circumference [HC]), and feeding methods during the first months of life (use of nasogastric tube at birth and duration of breastfeeding).

Growth and body composition were assessed around 1 year before gastrostomy (T0), up to 3 months before gastrostomy (T1), and around 1 year after gastrostomy (T2). Anthropometric measurements (weight and length) were obtained using a portable digital pediatric scale (Confort Welmy) and a portable stadiometer with an accuracy of 1 cm in the lying-down position. In addition, motor function was assessed 1 month before gastrostomy using the Gross Motor Function Classification System (GMFCS).

All assessments were performed by trained health professionals experienced in caring for children with CZS and trained in standardized measurement procedures. Additionally, these professionals were blinded to the purpose of the study, and the measurements used were part of the routine assessments performed at the rehabilitation center.

Absolute values of weight and length, body mass index (BMI), and anthropometric indicators (weight-for-age, height-for-age, BMI-for-age, and HC-for-age) were calculated using the Anthro^®^ software (version 3.2.2) and Anthro^®^ Plus software (version 1.04), proposed by the World Health Organization (WHO). Regarding weight-for-age and height-for-age, children were classified as very below expected (<-3), below expected (between −2 and −3), adequate (between 2 and −2), and above expected (>2), considering WHO growth reference curves^14^. Based on HC-for-age at birth, children were classified as without microcephaly (<-2), with mild microcephaly (between −2 and −3), severe microcephaly (<-3), and macrocephaly (>2), according to Intergrowth-21st growth reference curves^15^.

GMFCS is an ordinal scale to classify the mobility and functionality of children with cerebral palsy from level I (minimal limitations) to V (severe limitations)^16^.

For body composition, body fat was calculated using the Slaughter equation specific to cerebral palsy^17^. The triceps and subscapular skinfold thickness were measured using a caliper (Beta Technology, Santa Cruz, California) for this equation. Considering that the equations and corrections^17^ were based on skin color (white or black), the body fat was considered as the mean values of the equations proposed for White and Black individuals since the Brazilian population has predominantly mixed color.

### Statistical analysis

The sample was characterized according to the descriptive statistics, considering mean and standard deviation for continuous variables and absolute and relative frequency for categorical variables.

Absolute and relative frequency were also calculated for anthropometric measures of children with adequate weight, height, and BMI during assessments. The frequency of children in each classification of anthropometric measures was compared using the chi-square test before and after gastrostomy. Next, the Shapiro-Wilk test verified the normality of these measures and body fat values, and normal data were analyzed using analysis of variance (ANOVA) with repeated measures. When necessary, univariate analyses, Tukey's post hoc tests were employed. Additionally, effect sizes (η^2^) were calculated and classified as small (η^2^<0.06), medium (0.06≤η^2^<0.14), or large (η^2^>0.14). All analyses were performed using the Jamovi software (the Jamovi project [2020], version 2.3.28), with statistical significance set at 5%.

## RESULTS

A total of 23 children underwent gastrostomy; one died in the year following the procedure, one did not have pre-gastrostomy data available, and three were not followed up at the IPESQ. Thus, 18 children (10 boys and 8 girls) participated in the study, all classified at level V of the GMFCS, with ages between 20 and 75 months at the time of the gastrostomy (mean age of 47.2±18.7 months). After gastrostomy, all children were fed exclusively through this method due to severe stomatognathic disorders. T0 and T1 assessments were conducted 394.5±88.6 and 42.6±24.2 days before gastrostomy implantation, respectively. The T2 assessment was conducted approximately 376±65 days after the procedure. Table 1 presents the children's characteristics during pregnancy and at birth.

**Table 1:** Gestational and birth characteristics of children with congenital Zika syndrome undergoing gastrostomy (n=18), Paraíba, Brazil (April 2019 and May 2023).

Characteristics			n (%)	Mean±SD	Range
Caregivers’ characteristics					
	Age at child's gastrostomy			29.3±6.4	19.0–41.0
	Total family income (US$)**			369.9±169.3	156.1–696.4
	Per capita income (US$)**			91.7±35.5	34.1–160.2
Schooling					
	Elementary school		9 (50.0)		
	High school		9 (50.0)		
Steady partner					
	Yes		11 (61.1)		
	No		7 (38.9)		
Pregnancy characteristics					
	Symptoms of Zika virus infection				
		In the first trimester	8 (44.4)		
		In the second trimester	7 (38.9)		
		In the third trimester	1 (5.7)		
		No symptoms	2 (11.1)		
Duration of symptoms (days)				3.5±2.3	7–32
Gestational age (weeks)				38.6±2.03	32–40
Characteristics of the children at birth					
	Prematurity				
		Yes	2 (11.1)		
		No	16 (88.9)		
Breastfeeding in the first days of life					
	Yes		16 (88.9)		
	No		2 (11.1)		
Exclusive breastfeeding (days)				124.0±102.0	6–365
	Use of nasogastric tube at birth				
		Yes	5 (27.8)		
		No	13 (72.2)		
Length at birth (cm)				46.6±2.7	42–51
Weight at birth (kg)				2.8±0.5	1.8–3.7
HC at birth (cm)				29.7±1.9	26–33
Microcephaly at birth*					
	Yes		14 (82.4)		
	No		3 (17.6)		

SD: standard deviation; HC: head circumference; kg: kilograms; cm: centimeters.

* Medical records did not present anthropometric data for one child.

** Values were calculated considering the exchange rate of 1 US dollar=5.83 Reais.

Before gastrostomy, at T0, nine children had adequate weight, eight had adequate length, and seven had adequate BMI. At T1, five children had adequate weight, nine had adequate length, and six had adequate BMI. One year after gastrostomy, weight-for-age increased in 14 children (mean change of 1.38), length-for-age increased in 10 children (mean change of 0.75), and BMI-for-age increased in 13 children (mean change of 1.99). Following these changes, nine children were classified as adequate weight, nine as adequate length, and six as adequate BMI (Table 2). Table 3 presents the results of anthropometric indicators for each child at T0, T1, and T2.

**Table 2:** Anthropometric indicators of children with congenital Zika syndrome before and after gastrostomy (n=18), Paraíba, Brazil (April/2019 and May/2023).

Variables		T0	T1	T2	p-value	η^2^
Body weight (kg), mean±SD		9.8±2.^3^ a	10.9±2.^9^ a,b	14.2±3.^8^ b	<0.001	0.274
Weight-for-age, mean±SD		-2.7±1.2	-3.0±1^.^3f	-2.0±1.^9^ f	0.007	0.071
Weight classification, n (%)						
	Adequate	9 (50.0)	5 (27.8)	9 (50.0)		
	Below expected	1 (5.6)	3 (16.7)	2 (11.1)		
	Very below expected	8 (44.4)	10 (55.6)	7 (38.9)		
Length (cm), mean±SD		85.7±10.4^c^	92.4±11.9^c^,^d^	99.3±11.4^d^	<0.001	0.206
Length-for-age, mean±SD		-2.1±1.4	-2.3±1.4	-2.0±1.7	0.626	0.006
Length classification, n (%)						
Adequate		8 (44.4)	9 (50.0)	9 (50.0)		
Below expected		6 (33.3)	4 (22.2)	5 (27.8)		
Very below expected		4 (22.2)	5 (27.8)	4 (22.2)		
BMI, mean±SD		13.4±1.2	12.8±1.^4^ e	14.5±3.^3^ e	0.021	0.104
BMI-for-age, mean±SD		-2.0±1.1	-2.4±1.3	-1.2±2.5	0.07	0.007
BMI classification, n (%)						
Above		0 (0.0)	0 (0.0)	2 (11.1)		
Adequate		7 (38.9)	6 (33.3)	6 (33.3)		
Below		9 (50.0)	6 (33.3)	5 (27.8)		
Very below		2 (11.1)	6 (33.3)	5 (27.8)		

SD: standard deviation; T0: around 1 year before gastrostomy; T1: up to 3 months before gastrostomy; T2: around 1 year after gastrostomy; kg: kilograms; cm: centimeters; BMI: body mass index;

a-f the same letter indicates significant differences between assessments (p<0.05).

**Table 3:** Individual data of anthropometric indicators of children with congenital Zika syndrome before and after gastrostomy (n=18), Paraíba, Brazil (April 2019 and May 2023).

ID	Assessment	Days before and after gastrostomy	Body weight (kg)	Weight-for-age	Length (cm)	Length-for-age	BMI	BMI-for-age
1	T0	362	8.00	-1.84	70.0	-2.70	16.3	-0.30
	T1	17	8.75	-2.88	77.5	-3.37	14.6	-1.02
	T2	315	13.23	-0.56	87.0	-2.48	17.8	1.58
2	T0	533	9.77	-4.25	91.0	-3.82	12.0	-2.68
	T1	70	10.16	-4.87	100.0	-3.02	10.2	-4.60
	T2	385	11.33	-4.84	102.0	-3.70	10.9	-3.90
3	T0	380	11.51	-3.45	101.0	-1.96	11.4	-3.55
	T1	58	15.98	-1.75	106.0	-1.78	14.2	-0.86
	T2	237	15.77	-2.58	111.0	1.63	12.8	-2.31
4	T0	371	10.84	-1.63	92.0	0.05	13.0	-2.63
	T1	42	11.72	-2.01	95.5	-0.95	13.0	-2.21
	T2	484	21.92	1.52	103.0	-1.33	20.9	3.44
5	T0	360	10.21	-1.78	83.0	-2.03	15.1	-0.73
	T1	38	9.65	-3.33	87.0	-2.71	13.0	-2.39
	T2	354	14.18	-1.23	94.0	-2.54	16.3	0.74
6	T0	361	10.79	-1.7	90.0	-0.61	13.5	-2.07
	T1	24	11.74	-2.04	92.0	-1.93	14.1	-1.20
	T2	308	16.69	-0.09	98.0	-1.81	17.6	1.66
7	T0	439	12.20	-1.58	95.5	-0.77	13.6	-1.71
	T1	28	14.32	-1.35	108.0	0.39	12.4	-2.57
	T2	385	17.34	-0.84	117.0	0.90	12.7	-2.37
8	T0	261	6.44	-3.85	72.0	-2.96	12.4	-2.90
	T1	43	6.25	-4.99	71.0	-4.98	12.6	-2.69
	T2	349	7.47	-4.91	77.0	-5.15	12.8	-2.21
9	T0	216	7.15	-2.19	74.0	-0.69	13.1	-2.55
	T1	56	8.27	-1.93	79.0	-0.85	13.2	-2.04
	T2	365	10.26	-2.07	90.0	-0.92	12.9	-2.29
10	T0	359	9.45	-3.07	86.0	-2.22	13.0	-2.54
	T1	12	10.29	-4.01	95.0	-2.90	11.6	-3.45
	T2	492	11.25	-3.64	103.0	-1.60	10.7	-4.28
11	T0	442	5.43	-4.01	65.0	-3.30	12.9	-2.91
	T1	69	7.77	-3.27	72.0	-4.42	15.0	-0.33
	T2	410	11.62	-1.75	80.5	-4.45	18.2	-1.90
12	T0	428	8.31	-5.18	83.0	-5.45	12.3	-2.73
	T1	91	10.34	-4.62	94.0	-3.83	11.7	-3.45
	T2	476	11.35	-5.11	102.0	-3.65	10.9	-4.48
13	T0	362	13.81	-1.65	101.0	-1.42	13.7	-1.14
	T1	21	14.89	-1.85	107.0	-1.08	13.0	-1.73
	T2	371	18.21	-1.12	114.0	-0.85	14.0	-0.90
14	T0	374	8.04	-3.53	81.0	-2.14	12.3	-3.46
	T1	52	8.86	-3.79	86.0	-2.65	12.2	-3.30
	T2	320	16.22	0.07	92.0	-2.69	19.5	2.78
15	T0	393	12.99	-1.29	94.0	-1.73	14.9	-0.27
	T1	7	16.35	-0.55	105.0	-0.75	15.0	-0.16
	T2	378	20.70	0.31	108.0	-1.18	17.7	1.39
16	T0	484	12.82	-1.93	93.0	-2.77	15.0	-0.14
	T1	73	12.35	-3.23	102.0	-1.94	11.9	-2.87
	T2	425	13.74	-3.34	109.0	-1.93	11.6	-3.17
17	T0	372	8.95	-1.84	82.0	-0.19	13.3	-2.56
	T1	15	8.45	-3.79	87.0	-1.65	11.3	-4.35
	T2	356	10.31	-3.25	91.0	-2.43	12.6	-2.55
18	T0	604	10.50	-3.43	88.5	-3.73	13.6	-1.25
	T1	50	10.87	-4.30	100.0	-2.70	10.9	-3.88
	T2	356	13.82	-3.36	109.0	-2.02	11.6	-3.11

T0: around 1 year before gastrostomy; T1: up to 3 months before gastrostomy; T2: around 1 year after gastrostomy; BMI: body mass index; kg: kilograms; cm: centimeters.

The body fat before gastrostomy ranged between 19.9 and 41.1% at T0 (mean 32.7±6%) and between 20 and 32.9% at T1 (mean 25.2±3.8%). After gastrostomy, body fat ranged between 18.9 and 44.5%, with a mean of 29.3±6.5%. Statistical differences were observed between assessments (p<0.001, η^2^=0.24). Post hoc tests revealed differences between T0 and T1 (p<0.001) and between T1 and T2 (p=0.017).

## DISCUSSION

Although previous studies have shown a high incidence of weight below expected in children with CZS^10,18^, the impacts of alternative feeding methods, such as gastrostomy, have not been described for this population. Despite the significant increase in absolute values of weight, length, and BMI observed after gastrostomy in the present study, some children with CZS remained with BMI below expected and weight below or very below expected.

The increased mean weight after gastrostomy corroborated previous studies involving children with cerebral palsy^19,20^. However, some children with CZS remained with a weight below or very below expected 1 year after gastrostomy in this study, suggesting insufficient weight gain. Some hypotheses might explain this finding, such as the multiple comorbidities often experienced by children with CZS and inadequate caloric intake.

Jadi et al.^21^ described factors related to successful weight gain after gastrostomy in children with cerebral palsy. Although children with moderate and severe nutritional conditions showed improved results after 3 months, comorbidities (e.g., epilepsy, severe motor and muscle changes, and congenital gastrointestinal malformations) negatively impacted weight gain after 12 months (Jadi et al., 2023). Thus, the number of children with CZS who remained with weight below expected even 1 year after the gastrostomy may be justifiable since most children had severe motor impairments (i.e., level V of the GMFCS) and may have a high incidence of uncontrolled seizures^22^. However, information regarding comorbidities (e.g., occurrence of seizures) during the follow-up period and use of a nasogastric tube before gastrostomy was not collected, hampering the confirmation of this hypothesis. Also, congenital gastrointestinal malformations associated with intrauterine ZIKV infections cannot be excluded as the cause of these findings since the natural history of CZS had not been fully elucidated.

Considering that previous studies have shown that most children with CZS live in social and financial vulnerability conditions with limited access to basic health and sanitation^23,24^, some children might have received insufficient caloric intake for weight gain. Also, the present study did not corroborate the longitudinal study by Frawley et al.^25^ describing that exclusive gastric feeding was associated with reduced chances of underweight in children with CZS. However, these authors^25^ grouped children who used gastrostomy and nasogastric, orogastric, or nasojejunal tubes, limiting comparisons.

The body composition of children with CZS increased 1 year after gastrostomy, corroborating findings on children with cerebral palsy^26^. The body fat increase may be explained by the imbalance between caloric intake and energy expenditure previously described in children with severe neurological impairments^27^. Also, the reduced motor function in this population associated with excessive nutrient consumption may result in the storage of body fat^28^. The increased intake of ultra-processed foods and a limited variety of foods offered to children with CZS may also be related to the increased body fat^24^. Thus, these hypotheses and the fact that some children remained with a weight below expected 1 year after gastrostomy emphasized the need for nutritional monitoring to improve growth and development.

The present study aimed to understand the natural history of CZS, strengthened by its longitudinal follow-up design. However, some limitations need to be considered. The first is the small sample size. Although impairments in anthropometric parameters have been described in children with SCZ, caregivers of these children often fear gastrostomy implantation^10^, which may have contributed to the limited sample size in this study. Second, the study did not analyze comorbidities, eating habits (e.g., nutritional intake from artisanal or formula feeding, dysphagia diagnosis, use of anticonvulsants, occurrence of seizures, and hospitalization during the follow-up period) or post-gastrostomy dietary care, hygiene, and nutritional guidance. These factors could have influenced the nutritional status of children. This type of limitation is common in studies whose data source is medical records, particularly open cohorts such as the one described here.

One aspect that should be considered with caution in the present study is the measurement of the length. In the present study, length was measured in the lying-down position using a portable stadiometer, which may not be the most accurate method for children with severe motor impairment, who frequently present scoliosis and/or joint contractures^29^. During the study period, this method was used as a reference by the professionals from the center, who were trained to maintain the best children's alignment during measurement. However, in cases of significant scoliosis or joint contractures, length measurements were not performed.

Despite these limitations, the present study highlighted that some children with CZS remained with a weight below expected 1 year after gastrostomy and the need for future studies to investigate factors involved in the anthropometric results in this population.

The present study demonstrated that gastrostomy may increase weight, length, and BMI in children with CZS. However, this increase may be heterogeneous since some children remained with anthropometric measures below expected. Also, children with CZS showed an increase in body fat 1 year after gastrostomy.

## Data Availability

The datasets generated and/or analyzed during the current study are available from the corresponding author upon reasonable request.
